# Putative Repurposing of Lamivudine, a Nucleoside/Nucleotide Analogue and Antiretroviral to Improve the Outcome of Cancer and COVID-19 Patients

**DOI:** 10.3389/fonc.2021.664794

**Published:** 2021-07-21

**Authors:** José J. García-Trejo, Raquel Ortega, Mariel Zarco-Zavala

**Affiliations:** Department of Biology, Laboratory of Bioenergetics, Chemistry Faculty and School, National Autonomous University of Mexico (UNAM), Mexico City, Mexico

**Keywords:** Lamivudine, 3TC, cancer, COVID-19, radiotherapy, chemotherapy, SARS-CoV-2, RdRp RNA polymerase

## Abstract

Lamivudine, also widely known as 3TC belongs to a family of nucleotide/nucleoside analogues of cytidine or cytosine that inhibits the Reverse Transcriptase (RT) of retroviruses such as HIV. Lamivudine is currently indicated in combination with other antiretroviral agents for the treatment of HIV-1 infection or for chronic Hepatitis B (HBV) virus infection associated with evidence of hepatitis B viral replication and active liver inflammation. HBV reactivation in patients with HBV infections who receive anticancer chemotherapy can be a life-threatening complication during and after the completion of chemotherapy. Lamivudine is used, as well as other antiretrovirals, to prevent the reactivation of the Hepatitis B virus during and after chemotherapy. In addition, Lamivudine has been shown to sensitize cancer cells to chemotherapy. Lamivudine and other similar analogues also have direct positive effects in the prevention of cancer in hepatitis B or HIV positive patients, independently of chemotherapy or radiotherapy. Recently, it has been proposed that Lamivudine might be also repurposed against SARS-CoV-2 in the context of the COVID-19 pandemic. In this review we first examine recent reports on the re-usage of Lamivudine or 3TC against the SARS-CoV-2, and we present docking evidence carried out *in silico* suggesting that Lamivudine may bind and possibly work as an inhibitor of the SARS-CoV-2 RdRp RNA polymerase. We also evaluate and propose assessment of repurposing Lamivudine as anti-SARS-CoV-2 and anti-COVID-19 antiviral. Secondly, we summarize the published literature on the use of Lamivudine or (3TC) before or during chemotherapy to prevent reactivation of HBV, and examine reports of enhanced effectiveness of radiotherapy in combination with Lamivudine treatment against the cancerous cells or tissues. We show that the anti-cancer properties of Lamivudine are well established, whereas its putative anti-COVID effect is under investigation. The side effects of lamivudine and the appearance of resistance to 3TC are also discussed.

## Introduction

Drug repurposing takes advantage of previously FDA-approved drugs indicated for a particular illness, and focuses on using these drugs to improve the outcome of patients suffering a different disease. It is a viable option to treat several illnesses such as cancer, bacterial infections, and viral infections. Reexamining the properties of existing drugs is faster than *de novo* design and synthesis of new drugs. In addition, the safety profile is often well known, possibly making subsequent approval processes easier and more cost-effective. This is illustrated by some non-steroidal anti-inflammatory drugs (NSAIDs) which have recently been shown to improve the treatment of cancer patients receiving chemotherapy [reviewed in (1)]. For instance, Celecoxib (2) and related NSAIDs were found to increase the efficacy of cisplatin, paclitaxel, and doxorubicin against human cervical cancer (3). In addition, some anti-cancer drugs have been successfully used as efficient anti-bacterial agents (4), and as reviewed here, nucleoside/nucleotide analogues, have been repurposed as anti-cancer agents.

Nucleoside/nucleotide analogues such as Lamivudine, are a family of DNA and RNA polymerases inhibitors. These competitive inhibitors of DNA or RNA polymerases vary in their design and usage. Antivirals are generally classified based on their viral targets. The latter can be the viral structural components responsible for viral recognition and association to the host receptors in the cells, the viral components for entrance into the cells, and the viral nonstructural proteins that control the replication of the viral genome and ultimately the assembly process of the full viral particles. Several FDA-approved antiviral drugs are currently in use for viral illnesses such as HIV (AIDS), HBV, and HCV (Hepatitis B and C viral infections), Ebola, and more recently SARS-CoV and SARS-CoV-2. These include non-nucleotide analogue inhibitors of the protease that controls the entrance of the viruses into host cells, and the nucleoside/nucleotide analogues that inhibit the replication of the viral genetic material.

In this review, we will focus on nucleoside/nucleotide analogues first designed as anti-HIV, anti-Hepatitis, and anti-Ebola treatments and explore their repurposing in cancer treatment, and also as a putative repurposed antiviral in the fight against SARS-CoV-2. First we will explore the role of nucleoside/nucleotide analogues to improve the effectiveness of some chemotherapies against the growth of cancerous cells, with little or no effects in normal non-neoplasic cells. In particular, we will focus on Lamivudine (3TC), which was designed as an anti-HIV inhibitor, and we will explore its anti-neoplasic properties. We will also discuss its recently proposed repurposing against the SARS-CoV-2 and COVID-19 and we present docking experiments carried out *in silico* suggesting that Lamivudine may bind and possibly work as an inhibitor of the SARS-CoV-2 RdRp RNA polymerase.

## Properties of Nucleoside/Nucleotide AnalogUEs and Their Original Usage

Antiviral Nucleoside/Nucleotide Analogs (here abbreviated as ANNAs) are modified nucleosides or nucleotides that compete with their natural substrates (dCTP, dGTP, dTTP or dATP) for viral DNA or RNA replicases. The first drugs of this kind were developed to treat HIV infections in the 80s and 90s and included Nucleoside Reverse Transcriptase inhibitors (NRTIs) such as Zidovudine (AZT), Didanosine (ddI), Zalcitabine (ddC), Stavudine (d4T), Lamivudine (3TC), Abacavir (ABC), and Emtricitabine ((–)-FTC), and Nucleotide reverse transcriptase inhibitors (NtRTIs) such as Tenofovir (5).

The antivirals most frequently used for HIV, HBV and HCV infections are shown in **Table 1** and include Lamivudine or 3TC among others that have been reviewed before (5, 21, 22). In the current COVID-19 pandemic, new ANNAs, such as Remdesivir, originally developed to treat Ebola virus infections, and Sofosbuvir, approved to treat Hepatitis C infection, gained attention due to their potential effectiveness against the SARS-CoV-2 coronavirus (23–27). In this review, we will focus on the repurposing of Lamivudine and related ANNAs against cancer and its putative repurposing against COVID-19.

ANNAs are usually classified by their antiviral properties, but not by their natural substrate (nucleoside or nucleotide) from which they were derived. Here we classify them depending on their parent nucleotide or nucleoside (**Table 1**) so we can easily search specific molecular structures and identify those which may be repurposed. In nature, nucleotide-binding proteins control several key biological processes, from bioenergetic enzymes, to DNA and RNA polymerases. So, the information provided in **Table 1** could help to identify nucleoside/nucleotide analogues as a putative inhibitors or antagonists of other molecular targets.

**Table 1:** Some antiviral nucleoside/nucleotide analogues classified according to the natural nucleotides/nucleosides they compete with to inhibit viral DNA/RNA polymerases.

**Nucleotide/Nucleoside (natural substrate)**	**Analogue**	**Abbreviation**	**First Commercial name/full name-[references]**
**CTP, Cytidine**	Lamivudine	3TC	Epivir/ (–)-β-L-3′-thia-2′,3′-dideoxycytidine (6, 7)
	Zalcitabine or didesoxicytidine	ddC	HIVId/2′,3′-dideoxycytidine (8, 9)
	Emtricitabine	FTC	Emtriva (Coviracil)/ (–)-β-L-3′-thia-2′,3′-dideoxy-5-fluorocytidine (10)
**GTP, Guanosine**	Abacavir	ABC	Ziagen/2-amino-6-cyclopropylaminopurin-9-yl-2-cyclopentene (10)
**TTP, Thymidine**	Zidovudine	AZT o ZDV	Retrovir/3´-azido-2,3´-dideoxythymidine (12, 13)
	Stavudine	d4T	Festinavir Zerit, Zerit XR/2′,3′-dideoxy-2′,3′-didehydrothymidine (14)
	Censavudine	BMS-9860001	Bristol-Myers Squibb (15, 16).
**ATP, Adenosine**	Remdesivir		Veklury ^®^/L-Alanine, N-((S)-hydroxyphenoxyphosphinyl)-, 2-ethylbutyl ester, 6-ester with 2-C-(4-aminopyrrolo(2,1-f)(1,2,4)triazin-7-yl)-2,5-anhydro-D-altrononitrile (17)
	Didadosine	ddI	Videx, Videx EC/2′,3′-dideoxyinosine (9)
	EFdA	EFdA	EFdA/4’-etinil-2-fluoro-2’-deoxiadenosine (18)
**UTP, Uridine**	Sofosbuvir		Sovaldi ^®^/GS-5816 (19, 20)
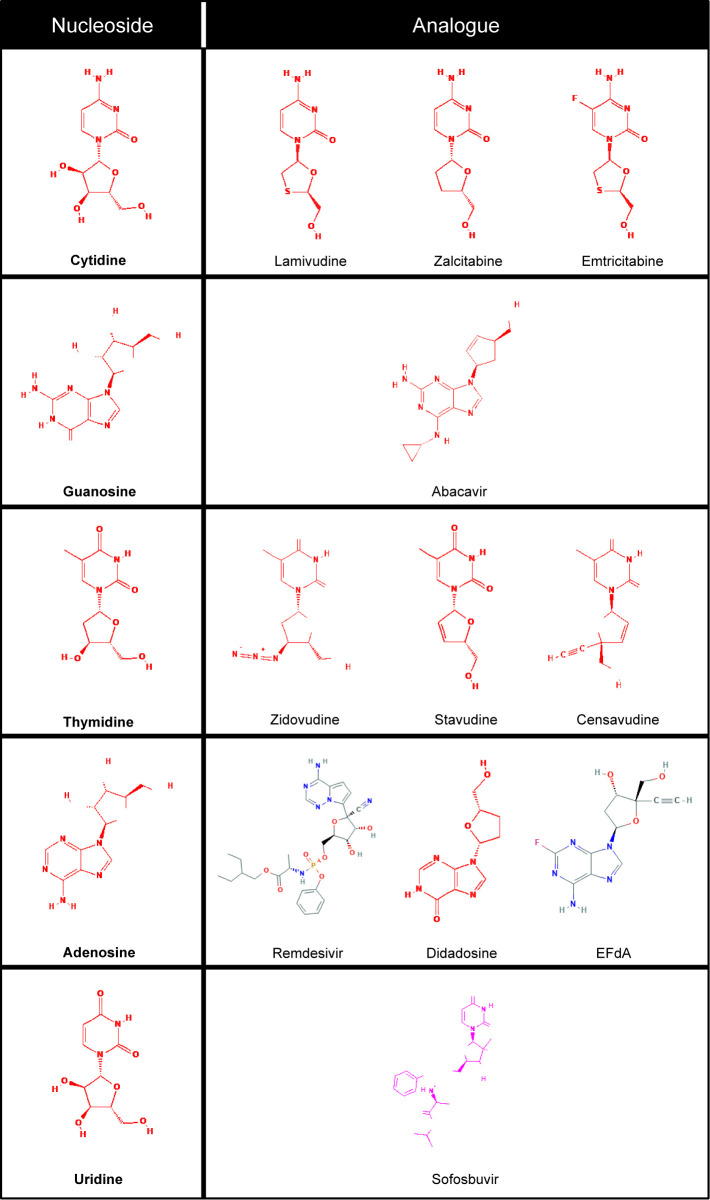					

This table was constructed from the information in references (5–20) and from the NCBI-Invertox Data base (NCBI-Invertox). https://www.ncbi.nlm.nih.gov/books/NBK548938/.

## Common and Distinctive Features on the Mechanism of Action of Antiviral Nucleoside/Nucleotide Analogues

**Figure 1:**
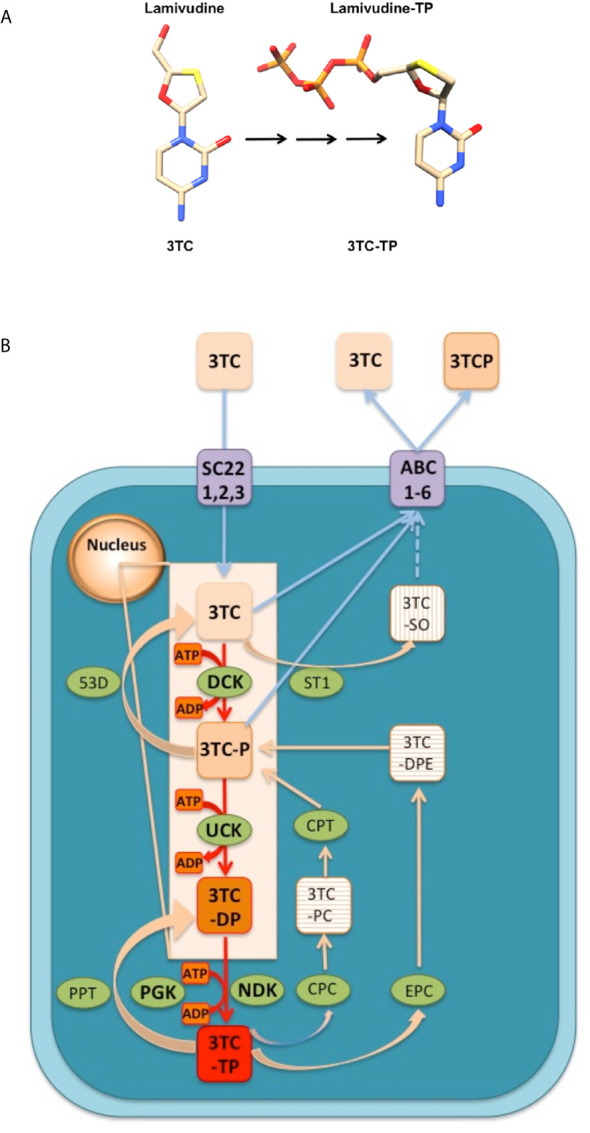
Conversion an ANNA in *vivo* from its pro-drug (Lamivudine) to the active triphosphate form (Lamivudine-TP). **(A)** The example of Lamivudine is shown and similar conversions take place for the other ANNAs. Left: Lamivudine in its pro-drug form. Right: Lamivudine triphosphate (Lamivudine-TP). Color codes: C, cream; N, blue; S, yellow; O, red; P, Orange. H´s are omitted for simplicity. The black arrows indicate three phosphorylation steps shown in red in **(B)**. Constructed in Chimera. **(B)** Transport and metabolism of Lamivudine (3TC) inside the cells. 3TC is the pro-drug that may be transported inwards (blue arrows) through passive diffusion or active transporters SLC22A1, SLC22A2, and SLC22A3 (SC22, 1,2,3). In the nucleus, 3TC is phosphorylated sequentially (red arrows) by Deoxycytidine kinase (DCK) to form Lamivudine-monophosphate (3TC-P) and by UMP-CMP kinase (UCK) to form Lamivudine-diphosphate (3TC-DP). In the cytosol, Lamivudine-diphosphate (3TC-DP) is phosphorylated by Nucleoside diphosphate kinase A (NDK) or by Phosphoglycerate kinase (PGK) to form Lamivudine-Triphosphate (3TC-TP). These three phosphorylation steps involve the dephosphorylation of ATP to form ADP (orange boxes). The high ATP/ADP ratio inside the cells drives the overall reaction “forward” in the sense of Lamivudine-TP or 3TC-TP formation (red box). 3TC-TP is the functional inhibitor of the viral (HIV and HBV) Reverse Transcriptase. On the other hand (left reactions) 3TC-TP and 3TC-P may be dephosphorylated by phosphatases (PPT), and by 5’(3’)-deoxyribonucleotidase (53D) to form 3TC-DP and 3TC, respectively, in “reverse” reactions. 3TC-TP may be metabolized (right side reactions) by Choline-phosphate cytidylyltransferase A (CPC) or by Ethanolamine-phosphate cytidylyltransferase (EPC) to form Lamivudine-phosphate-choline (3TC-PC) or Lamivudine-diphosphate-ethanolamine (3TC-DPE), which are transformed to lamivudine-phosphate (3TC-P), with the former reaction catalyzed by Cholinephosphotransferase 1 (CPT) in the inner membranes of the Golgi apparatus. The extrusion of Lamivudine (3TC) and Lamivudine-phosphate (3TC-P) (blue outward arrows), can be achieved actively by one to six different multidrug resistance proteins or ABC transporters (ABC, 1–6). On the other hand, Lamivudine or 3TC may also be transformed to Lamivudine sulfoxide (3TC-SO) by Sulfotransferase 1A1 (ST1), and 3TC-SO may be extruded by ABC transporters (blue discontinuous arrow). This figure was constructed according to the metabolism of Lamivudine as described in the references on the pharmacokinetics of lamivudine (28–31), and from the link of metabolism and action pathway (https://smpdb.ca/search?q=Lamivudine). Other ANNAs are metabolized similarly from the prodrug to the functional triphosphate form. See text and the link for details.

Generally, ANNAs are not prescribed as functionally inhibitory triphosphate forms, but as nucleoside or nucleotide pro-drugs which lack or replace the pyrophosphate moiety of the nucleoside triphosphates. The transformation of ANNAs pro-drugs to their functional triphosphate forms (**Figure 1A**), takes place in the nucleus and the cytosol, through the action of three consecutive phosphorylations catalyzed by intracellular kinases (28) as indicated in **Figure 1B**. The formation of the functional triphosphate form of Lamivudine (3TC-TP) can be used as an example to show how these ANNAs are transformed into their functional molecules (**Figure 1**). This process has been reported in several studies on the pharmacokinetics of Lamivudine (28–31), and summarized on line in the following link (https://smpdb.ca/search?q=Lamivudine) and on **Figure 1**. The pro-drug Lamivudine (3TC) is transported into the cell through active transport by SLC22A1, SLC22A2, and SLC22A3 (SC22, 1,2,3 in **Figure 1B**) or by passive diffusion (inward blue arrows in **Figure 1B**). Once inside the cells, Lamivudine (3TC) reaches the nucleus, where it is sequentially phosphorylated by Deoxy-Cytidine Kinase (DCK) to form Lamivudine-monophosphate (3TC-P) and then by UMP-CMP Kinase (UCK) to form Lamivudine-diphosphate (3TC-DP) (**Figure 1B**, red arrows in the nucleus). Finally, Lamivudine-diphosphate (3TC-DP) is phosphorylated in the cytosol by Nucleoside Diphosphate Kinase A (NDK) or by Phospho-Glycerate Kinase (PGK) to form Lamivudine-Triphosphate (3TC-TP). These three phosphorylation steps involve the dephosphorylation of ATP to form ADP (**Figure 1B**, orange boxes) to transfer sequentially the γ-phosphate from three ATP molecules to the monophosphate (3TC-P), diphosphate (3TC-DP), and triphosphate forms of Lamivudine (3TC-TP), respectively. The high ATP/ADP ratio inside the cells drives the overall reaction “forward” towards Lamivudine-TP (3TC-TP) formation. This triphosphate 3TC-TP competes with dCTP at the nucleotide binding site of the viral (HIV and HBV) Reverse Transcriptase enzyme, resulting in functional inhibition. In the reverse reaction, 3TC-TP and 3TC-P may be dephosphorylated by phosphatases (PPT), and 5’(3’)-deoxyribonucleotidase (53D) to form 3TC-DP and 3TC, respectively, (left side reactions of **Figure 1B**). Alternatively, 3TC-TP may be also reverse-metabolized (right side reactions in **Figure 1B**) by Choline-phosphate cytidylyltransferase A (CPC) or by Ethanolamine-phosphate cytidylyltransferase (EPC) to form Lamivudine-phosphate-choline (3TC-PC) or Lamivudine-diphosphate-ethanolamine (3TC-DPE), which are then reverse-metabolized to Lamivudine-phosphate (3TC-P), with the former reaction catalyzed by Cholinephosphotransferase 1 (CPT) in the inner membranes of the Golgi apparatus (not shown in **Figure 1B** for simplicity). It is worth noting, however, that these side reactions of Lamivudine and their metabolites have a slower metabolic flow due to the intracellular high [ATP] concentrations relative to [ADP], which drive “forward” the synthesis of 3TC-TP (red arrows in **Figure 1B**). The synthesis of Lamivudine-TP (3TC-TP) also competes with the active outward transport of Lamivudine (3TC) and Lamivudine-phosphate (3TC-P) to release them out of the cell (**Figure 1B**, outward blue arrows), carried out by up to six different multidrug resistance proteins or ABC transporters (ABC, 1–6). On the other hand, Lamivudine or 3TC may also be transformed into Lamivudine sulfoxide (3TC-SO) by Sulfotransferase 1A1 (ST1), and 3TC-SO may also be extruded by ABC transporters, although this has not been yet demonstrated (discontinuous arrow in **Figure 1B**). However, extrusion of 3TC-SO by the ABC transporters is suggested by the fact that Lamivudine sulfoxide is known to be removed through the kidneys. The final functional form, Lamivudine-triphosphate (3TC-TP) inhibits the Reverse Transcriptase of HIV or HBV viruses, to block viral replication by chain termination. Similar metabolic reactions drive the formation of the functional triphosphate forms of other ANNAs to form the functional triphosphate forms of these molecules. For further details readers may see the references cited (28–31) from which the metabolism of Lamivudine and its pharmacokinetics has been resumed here, and the SMPDB link (https://smpdb.ca/search?q=Lamivudine).

This process of transformation of the ANNAs pro-drugs to their triphosphate functional forms has an unknown efficiency yield, although the pharmacokinetics of Lamivudine and related ANNAs in healthy controls or HBV patients shows a measurable concentration of the pro-drug in the human plasma (28–31). The actual concentrations of the active TP-forms of the ANNAs within the cells are unknown. As described above, a fraction of the ANNAs may be lost or accumulated and further released through the kidneys as sulfonate modified non-metabolized intermediates (**Figure 1B**). As a result, only a fraction of the administered pro-drugs is effectively transformed into the inhibitory triphosphate forms and are able to reach the target viral enzymes in the cytoplasm of the cells. In the patients suffering from HIV or viral Hepatitis, ANNAs only partially inhibit the viral replication. Thus, the patients need to adjust the doses of ANNAs to keep the viral replication as low as possible. If the ANNAs treatment is interrupted, replicative capacity is likely to be restored and the patients may suffer from an increase in viral load. In consequence, it is crucial to find the right dosage of the ANNAs to keep the viral load as low as possible in a latent state in these patients, while preventing the side effects of the ANNAs.

**Figure 2:**
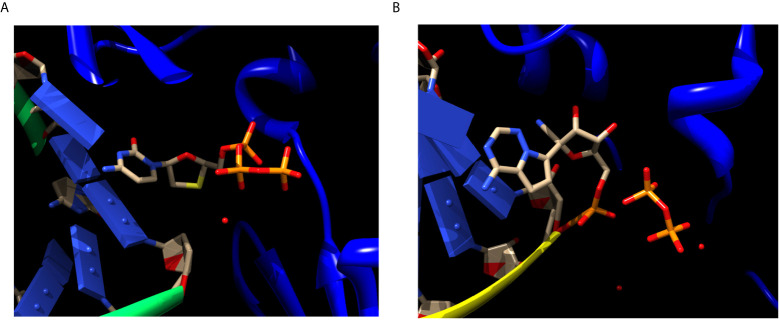
Chain termination inhibition by Lamivudine and delayed chain termination inhibition by Remdesivir of viral RNA polymerization. **(A)** Structure of the Reverse transcriptase (blue ribbons) of hepatitis virus complexed with RNA (green ribbons/blue domino-like bases) and Lamivudine or 3TC (sticks). Lamivudine is not yet, but about to be incorporated in to the newly synthesized DNA chain, thus preserves its triphosphate moiety. The ribose of Lamivudine lacks both 2´-OH and 3´-OH, in yellow the sulfur atom replacing the C3´-OH. The lack of both the 2´-OH and 3´-OH inhibits the processive DNA polymerization immediately after being incorporated into the nascent DNA. The lack of the 2´-OH inhibits the proof reading activity of the polymerases. Source: PDB_id 6KDJ. **(B)** Structure of the SARS-CoV-2 RdRp polymerase (blue ribbons) complexed with RNA (yellow ribbons/blue domino-like bases) and Remdesivir-MP (sticks). The Remdesivir-MP is covalently linked to the nascent RNA chain through its α-phosphate, and the pyrophosphate (PPi) released from its incorporation is shown (sticks). The ribose moiety of Remdesivir contains both the 2´-OH and the 3´-OH (pointing “up” in the ribose), thus allowing its incorporation into the newly synthesized RNA for about four to five additional bases. Subsequently, the RNA polymerization is stalled by steric hindrances imposed by the nitro substituent of Remdesivir. Source: PDB_id 7BV2. This explains why this mechanism is called “delayed” chain termination, because it allows incorporation of additional four to five additional bases. Only the ANNAs binding sites are shown for clarity, the rest of the proteins, backbones, residues and other ligands are excluded. The red spots are water molecules. Atom color codes in sticks are the same as in **Figure 1**. Figures constructed in Chimera.

Like lamivudine, the functional triphosphate forms of most nucleoside/nucleotide analogues work by a similar “chain termination” dead-end inhibitory mechanism, in which the processive activity of the viral DNA polymerase, is literally terminated. Termination could either occur due to the lack of an essential 3’-OH in the ribose structure, or by the addition of a bulky substituent group that leads to steric hindrances in the processive mechanism of nucleic acid polymerization of these enzymes (**Figure 2**). In the first example, nucleoside/nucleotide analogues are incorporated into the nascent RNA or DNA molecule as the natural substrates, but due to the lack of the 3´-hydroxyl group in most of these analogues, polymerization is halted or terminated immediately. Examples of these instantaneous chain terminators include Lamivudine and Zidovudine (32). Some more recently developed nucleoside/nucleotide analogues such as Remdesivir, are delayed chain terminators. These inhibitors have both hydroxyl groups in the ribose, 2´ and 3´, so they allow the formation of the chain phosphodiester bond, but the presence of a nitro group causes the delayed-chain termination by finishing the polymerization in the position n+4 or n+5 after the incorporation of the nucleotide analogue in the nascent RNA chain (23) (see **Figure 2**).

While the chain termination effects of ANNAs are well-known, most ANNAs are not specific to viral DNA polymerases. Many, if not all of them interact with their target enzymes and also with other nucleotide-binding proteins, This lack of specificity opens the door to repurposing ANNAs for other therapeutic purposes. In addition to the functional triphosphate form of ANNAs, studies of drug repurposing have predicted that ANNAs and other drugs may bind to surface viral proteins and could interfere with viral entrance through the cell membranes (33–35). In summary, the lack of total specificity of these nucleoside/nucleotide analogues is the source of the heterogeneous effects that might allow the repurposing of these drugs and their pro-drugs in other viral illnesses and oncogenic processes.

## Repurposing Nucleoside/Nucleotide Antivirals in Cancer

As described above, nucleoside/nucleotide analogues, designed initially as antivirals, have been found useful relatively recently for re-usage against other illnesses. Some researchers have found that these ANNAs can be re-used against a new set of viruses different to the original targets. For instance, some antivirals such as Remdesivir or Sofosbuvir originally designed to inhibit the viral Reverse transcriptase or RNA-dependent-RNA polymerase (RdRp) from viruses like Ebola or HIV, have been proposed (25, 26, 36, 37) and shown (38) to inhibit the SARS-CoV-2 RdRp RNA polymerase. This re-usage within the viral field is relatively common since the RT DNA polymerases and RdRp RNA polymerases are homologous enzymes in retroviruses or RNA viruses. These enzymes conserve the general and characteristic “right hand” architecture of the so-called palm, thumb, and fingers domains, and keep the catalytic site that binds the same or similar nucleotides as substrates. Thus, it is not unexpected that some ANNAs designed for instance against Ebola or HIV viruses might work against other RNA viruses like SARS-CoV, SARS-CoV-2 and so on. Indeed, Remdesivir seems like a promising therapy against the COVID-19 illness and it has been approved by the FDA as a clinical therapy. A broader extension of this approach is the re-usage of ANNAs in other fields like microbiology or cancer research, where the secondary targets are not viral. For instance, it has been found that some ANNAs can work as antimicrobial agents against some bacteria (21).

The idea that ANNAs have a potential therapeutic re-usage against cancer is not new as from the beginning of their usage against viral infections, the researchers that synthesized these ANNAs realized that some human cancers are produced by viruses. For instance, Hepatitis B and C (HBV and HVC) has a strong association with Hepato-Cellular carcinoma (HCC); Epstein–Barr virus (EBV) with naso-pharingeal carcinoma (NPC); Burkitt´s lymphoma, (BL) non-Hodgkin B-cell lymphoma, which also occurs in response to immunodeficiency syndrome (AIDS), are associated with HIV virus; and Human papilloma virus (HPV) has a strong association with cervical carcinoma, among other virus-cancer associations. Therefore it was envisaged from the very beginning of ANNAs’ synthesis and antiviral applications that the use of ANNAs may help to prevent the development of human cancers associated with viral infections (39, 40). However, more recently, some research groups have reported that ANNAs may not only prevent the development of viral-associated cancers, but that they have a direct effect on neoplastic processes. These recent reports tested the effect of Zidovudine, Abacavir, and Lamivudine on the growth of cancerous cells *in vitro* (41). The rationale behind these assays is that most if not all cancerous cells, exhibit a shortening in the length of their telomeres and an increase in telomerase activity. When radiotherapy is used to treat cancer, it damages the DNA of cancerous cells and inhibits telomerase activity. Unfortunately, this therapy also affects normal non-cancerous cells. Telomerase, whose increased activity is necessary in cancer cells for the maintenance of their telomeres, has a reverse transcriptase (RT) activity in one of its structural subunits. Therefore, the researchers hypothesized that ANNAs originally designed as inhibitors of the viral reverse transcriptases (RTs), may also have a non-specific effect on telomerase which could support their use as an anti-cancer therapy. The researchers assay ANNAs in combination with radiotherapy of cancerous cells to test the hypothesis that the ANNAs could improve the sensitivity of the cancerous cells to radiotherapy by inhibiting the RT activity of the telomerase. In this context, Zhou et al. (42) studied the effect of Zidovudine on the radiosensitivity of human malignant glioma cells. Their results showed that Zidovudine exerted an increased radiosensitization by inhibiting the activity of the telomerase (42). At that time, it was not yet shown if other ANNAs such as Lamivudine and/or Abacavir could exert a similar radiosensitization effect in cancer cells, although it was already known that these two ANNAs inhibited *in vitro* the telomerase activity of peripheral mononuclear cells (43).

The next question was whether other ANNAs such as Lamivudine and/or Abacavir could exert a similar radiosensitization effect in cancer cells. Therefore, some other researchers such as Chen et al. (41), returned to this idea and assayed the effect of Zidovudine, Abacavir, and Lamivudine as radiosensitizers on human esophageal cancer cells, finding that preincubation with these three ANNAs increased the radiosensitivity of the esophageal cancer cells, through an increase in DNA damage by radiation, increased apoptosis, deregulation of telomerase activity, and by decreasing the length of the telomeres as induced by radiation (41, 42). These results support the hypothesis that ANNAs work as telomerase inhibitors and the resultant radiosensitization, could improve the outcomes in cancer patients exposed to radiotherapy.

Besides the putative repurposing of ANNAs as radiotherapy sensitizers, there is another practical advantage in the re-usage of these molecules in cancer patients. Unfortunately some patients with cancer also have viral hepatitis, which complicates their treatment with radiotherapy or chemotherapy. The latter treatments increase the risk of viral reactivation due to the immunosuppression. In these patients, ANNAs such as Lamivudine and others may be useful to radiosensitize the cancerous cells or tumors and to prevent the reactivation of viral replication, even *before* and not only *during* chemotherapy or radiotherapy. One group has tested the hypothesis of the re-usage of ANNAs to prevent the reactivation of viral replication during chemotherapy directly in the clinic in a cancer patient treated with chemotherapy (44). This was the case of an elderly male HBV patient suffering from prostate adenocarcinoma and bone metastases, treated by chemotherapy with mitoxantrone and prednisone. When HVB is reactivated as a complication of cytotoxic treatment, it can lead to subfulminant acute hepatitis which can eventually derive to fulminant liver damage. This case and others are examples of successful treatments of HBV positive cancer patients with Lamivudine and/or Adefovir where the viral presence became negative after the treatment compared with placebos (44). In summary, Lamivudine and/or Adefovir can help to treat and prevent hepatitis virus reactivation during chemotherapy, thus leading to a clear improvement in the outcome for HBV patients treated with chemotherapy.

Other recent reports on the prophylactic use of Lamivudine and other ANNAs during the treatment of cancer, include HBV patients with diffuse large B-cell lymphoma treated with rituximab-containing chemotherapy (45). In this case Lamivudine and Entecavir reduced the occurrence of HBV reactivation-related hepatitis and mortality (45). In this study, Entecavir exerted a more evident prophylactic effect than Lamivudine. Thus it seems possible that other ANNAs besides 3TC are useful in preventing viral reactivation and resistance to chemotherapy. Also, Lamivudine has been shown to enhance the sensitivity of liver cancer cell lines to chemotherapy with sorafenib, by decreasing partially the expression of an apoptosis inhibitor (cIAP2), thus diminishing the viability of liver cancerous cells (46). Moreover, in cultured cancerous cell lines from human breast cancer it was also found that Lamivudine, combined with other ANNAs such as Abacavir, Stavudine, and Tenofovir, significantly increased apoptosis and decreased migration more effectively in the cancerous cell lines than in normal breast cell lines (47). This opens the possibility for further research to find the optimal combinations of ANNAs to decrease viability and migration of particular types of cancerous cells and tumors.

Furthermore, new nanotechnologies have been applied to the delivery of Lamivudine to lung and skin cancerous cells. A recent study showed enhanced inhibition of proliferation of cancer cell lines by nanoparticle-encapsulated Lamivudine compared with the free Lamivudine (48). Other works had assayed some cysteine and arginine rich peptides (49) and multivalent tricyclic peptides (50) as vehicles to deliver Lamivudine into the target cells. In the first case, a number of cysteine and arginine containing linear and cyclic peptides were challenged to see whether these peptides could improve the intracellular transportation of two cargoes, a fluorescently-labeled and cell-impermeable phosphopeptide (F´-GpYEEI) and fluorescently-labeled Lamivudine (F-3TC). The study showed that among the different peptides, the cyclic peptide was more efficient in transporting the cargoes into the cells, enhancing transport 16–20-fold (49). Interestingly, the experiments were carried out in human leukemia cancer cells (CCRF-CEM), thus showing that this delivery procedure may be useful as a therapy against cancerous cells (49). In the second case, a trycyclic peptide was constructed with an optimal balance of positive charge and hydrophobicity to improve its interactions with the cell membranes and promote transport of the cargo; in this case fluorescently labeled anti-HIV drugs such as F´-Lamivudine (F´-3TC), F´-Emtricitabine (F´-TC), and F´-Stavudine (F´-d4T). In all cases, the trycyclic peptide used as molecular transporter enhanced the cellular uptakes of these fluorescently labeled pro-drugs (50). In summary, new delivery strategies are being optimized to improve the entrance of Lamivudine and other ANNAs into target cells (**Figure 1B**), which could support the repurposing of Lamivudine and other ANNAs to work as direct anti-cancer therapies *in vitro* and *in vivo*.

Finally, an extended 10 year (2007–2017) study was recently described analyzing the trend in antiviral treatment uptake comparing the effect of different ANNAs’ initiation times on survival, before and after hepatocellular carcinoma (HCC) diagnosis (51). The study included treating HCC patients with Lamivudine, Adefovir dipivoxil, Entecavir, Telbivudine, and Tenofovir. This study concluded that the uptake of ANNAs treatment in HCC patients increased over the past decade, and that ANNAs treatment improved survival. This was independent of whether the ANNAs treatment started before or after HCC diagnosis, and also independent of primary treatments. These results are significant because they show that Lamivudine and related ANNAs show an increase in patient survival. Furthermore, this increased survival is solely due the combination of ANNAs with radiotherapy or chemotherapy, and independent of the primary treatment (51).

Lamivudine has also been shown to prevent the development of Hepato Cellular Carcinoma (HCC). HBV patients often develop chronic cirrhosis, which increases the risk of HCC. Treatment with Lamivudine alone or in combination with other ANNAs, results in HBV patients being less likely to develop HCC. This was reported in single clinical studies (51) and in a recent systematic review and meta-analysis (52). In summary, along with the prevention of reactivation of HBV in cancer patients, Lamivudine and other ANNAs are useful as preventive therapy to avoid the development of HCC in chronic HBV patients by suppressing the development of chronic cirrhosis.

One last benefit of Lamivudine in cancer and radiotherapy has been reported in an HIV patient also suffering from MALT (Mucosa-Associated Lymphoid Tissue Type Lymphoma) (53). Because the patient showed a high viral load, it was treated with a combination antiretroviral therapy including Zidovudine, Lamivudine and Lopinavir/Ritonavir. The treatment decreased the viral load to near zero, the lymphoma was put into remission, and the symptoms were completely resolved in the first 16 weeks of ANNAs and Lopinavir/Ritonavir treatment. A residual tumor mass persisted and was resolved by 3 months of radiotherapy. The patient was well after 5 years of follow up. It was considered that the lymphoma could had been resolved by the combined antiretroviral therapy without radiotherapy, but the latter was applied to accelerate the resolution of the lymphoma. It is hard to know whether the Lamivudine/Zidovudine or the Lopinavir/Ritonavir combinations, or both, were the key factors resolving the lymphoma, but it shows that the right combinations of ANNAs and other antiviral therapies can increase the odds of a positive outcome. It is worth to point out that these positive effects of Lamivudine are not exclusive of a single type of cancer, because it worked well not only in cancer patients suffering from hepatoma or prostate cancer treated by radiotherapy or chemotherapy, but also in patients harboring a lymphoma, with the antiviral therapy working well, previous to the beginning of radiotherapy or chemotherapy.

These reports of *in vitro* and *in vivo* repurposing of Lamivudine and related ANNAs as adjuvants in anti-cancer therapies open the door to the possibility that ANNAs are a potentially helpful approach to improve the outcome of radiotherapy and/or chemotherapy anti-cancer treatments that usually are associated with undesirable broad side effects in the patients. Co-administration of ANNAs with radiotherapy or chemotherapy inhibits cancer cell growth by inactivating the telomerase, and/or by inducing apoptosis. Furthermore, co-administration of ANNAs with radiotherapy or chemotherapy may prevent the reactivation of hepatitis virus in HBV positive cancer patients, and possibly in other viral illnesses. Recently, it has been shown that some inhibitors of the viral main proteases such as Lopinavir and/or Ritonavir are also effective in the reduction of proliferation cancer cells in several kinds of tumors. The reduction in the growth of cancerous cells takes place by a mechanism different to those of repurposed ANNAs, since these other inhibitors do not inhibit the DNA or RNA polymerases, but seem to work on p53, CD receptors, and in a convergent way with ANNAs, by inducing apoptosis, but by still unknown mechanisms (54). Finally, while some combinations of chemotherapy and ANNAs, have recently been shown to promote a positive outcome, other combinations seem to have adverse effects. For instance, Gemcitabine enhanced the antiviral activities of Tenofovir, Abacavir and Emtricitabine; whereas Pemetrexed did not affect Tenofovir, enhanced Abacavir, but decreased Emtricitabine and Lamivudine (3TC) antiviral activities (55). In conclusion, future research on the different combinations with Lamivudine or other ANNAs is needed to ascertain the optimal ANNAs combination to improve the outcome of the patients of different types of cancer.

It is worth mentioning that this review summarizing these studies is not intended to conclude that Lamivudine is uniquely useful in cancer patients. Other ANNAs may be as good as, or even better than Lamivudine as a co-therapy to improve the outcome in cancer patients. For instance, it has been recently reported that another ANNA such as Entecavir (ETV, dGTP analogue) may have better efficacy than Lamivudine for the rescue of chemotherapy-induced HBV reactivation (56). Entecavir is an alternative ANNA that may be used to rescue chronic HBV or HIV patients from resistance to Lamivudine. Furthermore, Non-Nucleos(t)ide Anti-Retroviral Inhibitors (NNRTIs) have been shown to be a preferred co-therapy to improve the outcome of the AIDS-related Kaposi´s sarcoma according to a prospective cohort study (57). It seems therefore that other non-ANNAs antiretrovirals may also contribute to single or combined antiretroviral therapies (ARTs) to improve the outcome of cancer patients; however, the effect of non-ANNAs in cancer prophylactic therapies, are out of the scope of this review. In summary, the field is open to test the effects of other ANNAs or non-ANNAs, alone or combined with Lamivudine as prophylactic therapies in cancer patients.

## Other Repurposing or Putative Reusage of ANNAs and Lamivudine Against SARS-CoV-2 and COVID-19

As explained above, the ANNAs are non-specific for the viral DNA, RT or RNA polymerases thus other repurposing applications are appearing. For instance, antibacterial effects of some of these ANNAs (21) open the exciting window of future antimicrobial treatments. In the reminder of this review we assessed whether some ANNAs, besides Remdesivir, could be repurposed, from inhibiting replication of HIV or hepatitis viruses, to inhibiting the RdRp-RNA polymerase of the SARS-CoV-2. Some of these ANNAs have been shown to inhibit the RdRp of the SARS-CoV-2 *in vitro* (38). The most promising ANNA exerting this is the ATP analogue Remdesivir (see **Table 1**), whose inhibitory position and structure on the RdRp of the SARS-CoV-2 has been already resolved by cryo-EM. Similar ANNAs but UTP analogues such as Sofosbuvir have been proposed and used in clinical trials with promising results in COVID-19 patients (25, 26, 36, 37). These relatively new ANNAs were approved by FDA very recently, originally as anti-Ebola inhibitors. As stated above, Remdesivir was recently approved as anti-COVID-19 treatment; however, the OMS concluded recently that Remdesivir does not show a significant positive therapeutic effect on the time of hospitalization, recovery time, and number of deaths in COVID-19 patients [WHO (58)]. In addition, it is expensive and sometimes only available during clinical trials or government programs. Therefore, the need for better and more accessible antiviral drugs is a matter of high priority over and above the need for vaccines. Thus we reasoned that we should examine existing FDA-approved ANNAs that could also work as RdRp inhibitors of the SARS-CoV-2 coronavirus. These “vintage” ANNAs could be a source of less expensive and more accessible anti-COVID-19 therapies.

We disregarded ANNAs or non-ANNAs viral inhibitors that have already been formally proposed or under clinical anti-COVID-19 trials as anti-SARS-CoV-2 drugs, such as Remdesivir or Sofosbuvir. We found that Lamivudine (3TC) is not currently under COVID-19 clinical trials although it has a reported inhibitory effect on the RdRp-RNA polymerase of a related hepatitis virus (59). A single report has proposed Lamivudine or 3TC as a repurposed antiviral anti-SARS-CoV-2 but only as another option among many with no supportive data for 3TC as anti-COVID-19 therapy (24). Therefore, we carried out *in silico* modeling and docking analyses to test whether Lamivudine (3TC) in its inhibitory triphosphate form (Lamivudine-TP or 3TC-TP) could bind with higher affinity than the natural nucleotide CTP to exert chain termination of the SARS-CoV-2 RdRp. A possible argument against the repurposing of Lamivudine in RdRp dependent virus such as SARS-CoV-2 is that RdRps do not use dNTPs, but NTPs for processive RNA polymerization and vice versa, i.e., DNA and RT-DNA polymerases use dNTPs as substrates for DNA polymerization, but not NTPs. Thus, it could be argued that the SARS-CoV-2 RdRp may not bind 2´3´ddCTP analogs such as Lamivudine because it lacks both the 2´-OH and the 3´-OH hydroxyls in its ribose moiety (see **Table 1** and **Figures 1**, **2**). However, several arguments support the binding of 2´3´-ddNTPs to the viral RdRps that may result in chain termination. For instance, biophysical (60) and crystallographic studies (61, 62) have shown that ddCTP binds to the Polio virus RdRp in the catalytic site.

Also and remarkably it has been experimentally demonstrated that Lamivudine-TP (3TC-TP) is a strong inhibitor of the RdRp activity of the NS5B subunit of the hepatitis C virus (59). The RdRp-RNA polymerase of Polio virus (PV) and that of the NS5B Hepatitis C virus are “right hand” RdRp RNA polymerases, homologous to that of SARS-CoV-2. Therefore these antecedents strongly suggest that Lamivudine must be able to inhibit the SARS-CoV-2 RdRp. Recent reports show that Lamivudine-TP may (38) or may not (63) be able to inhibit and be incorporated into the nascent SARS-CoV-2 RNA chain *in vitro* or *in vivo*, respectively. In the first case (38), very precise mass spectrometry analyses showed Lamivudine-TP or 3TC-TP was incorporated into the nascent RNA by the SARS-CoV-2 RdRp or promoted the mis-incorporation of wrong nucleotide-TPs, thus either halting or producing mutations in the nascent SARS-CoV-2 RNA. In the latter case, it was found that Lamivudine or 3TC did not affect the SARS-CoV-2 RNA replication in monkey and human cancerous cultured cells (63), however it remains to be carried out in non-cancerous human cultured cells. We suggest that as shown before (64) human MRC5 lung epithelial cells might be an excellent experimental model, to explore further the effect of Lamivudine and other ANNAs in SARS-CoV-2 replication. In summary, there are opposing reports respecting inhibition of the SARS-CoV-2 RdRp by Lamivudine. Therefore more *in silico*, *in vitro*, and *in vivo* research is needed to confirm whether Lamivudine may or may not be effective against the SARS-CoV-2 replication. Lamivudine lacks the ribose 2´-OH hydroxyl which is essential for the expression of the proof-reading 3’-5’ exonuclease activity of the SARS-CoV-2 RdRp-RNA polymerase (65), therefore it should block this enzyme´s proof-reading activity by lacking the ribose 2´-OH, besides putatively exerting the RNA polymerization termination because it also lacks the ribose 3’-OH. The Lamivudine targets may extend from DNA or RT-DNA polymerases of retroviruses to the RNA-dependent-RNA-polymerase (RdRp), and this has been explained by the similarity among RT-DNA polymerases and RdRp RNA polymerases (66).

We assessed the re-usage of Lamivudine against COVID-19 *in silico* by docking Lamivudine into the cryo-EM structures of the SARS-CoV-2-RdRp catalytic chain. In the course of this work, two new cryo-EM structures of the SARS-CoV-2-RdRp resolved by cryo-EM were uploaded into PDB, including the current SARS-CoV-2-RdRp (PDB_id 6M71.pdb), which was used for direct docking, and the SARS-CoV-2-RdRp bound to template RNA and the inhibitor Remdesivir (PDB_id 7BV2.pdb), which was found bound in this structure in its monophosphate form (Remdesivir-MP) close to the pyrophosphate released from the original Remdesivir-triphosphate inhibitor (see **Figure 2**). The latter was used to find the functional binding site of the docked nucleoside analogue inhibitors of the SARS-CoV-2-RdRp in the catalytic site of the protein, and to select the closest Lamivudine/Lamivudine-TP that docked and overlapped with the observed Remdesivir-MP of the cryo-EM structure. This was carried out after structural alignment of the nucleoside analogue or nucleoside triphosphate docked to the 6M71.pdb (chain A), with the 7BV2.pdb (chain A). In this way, several docked NTPs and nucleoside/nucleotide analogue clusters were discarded as false positives along the surface of the protein or outside the catalytic site. All dockings were carried out with the SARS-CoV-2 RdRp chain A, i.e. the main catalytic subunit, removing the other accessory subunits before docking. These docking Nsp-12 targets were prepared for docking with the DockPrep tool of Chimera. All dockings were carried out with SwissDock (http://www.swissdock.ch/). This platform carries out docking by executing in general five steps, 1) identification of cavities in the putative binding sites of the protein; 2) generation of binding modes (BM) or poses of the ligand moving its dihedral angles; 3) scoring of data binding analysis taking into account electrostatic and Van der Waals interactions using the force field CHARMM 22; 4) refinement of resulting BM including energy minimization; and finally, 5) a clustering analysis to obtain the best BM. This procedure was carried out with flexible docking allowed only for the ligand and in the absence of Mg^2+^ since some ligands available from cryo-EM or crystallographic structures contained a bound Mg^2+^ ion. However, other ligands lacked Mg^2+^, therefore in order to carry out all dockings in the same conditions and without any bias, the dockings were carried out in the absence of Mg^2+^. Results from docking were analyzed and visualized in Chimera and PyMol on a Mac-Pro running under OS X 10.116 with a 3.7 GHz Quad-Core Inter Xeon E5 processor. Positive controls of the docking procedure were carried out by docking the Remdesivir triphosphate (Remdesivir-TP) extracted from the cryo-EM structure (PDB_id 7BV2.pdb) as Remdesivir-MP and constructed in Chimera as Remdesivir-TP, from the SARS-CoV-2–RdRp-RNA–Remdesivir complex structure, and prepared for docking into the apo-RdRp (PDB_id 6M71.pdb). Importantly, the chosen docked nucleotides were those overlapping with the Remdesivir-monophosphate (Remdesivir-MP) just incorporated into the nascent coronaviral RNA molecule, and not with the Remdesivir-MP that has advanced further four to five additional nucleotide incorporation steps, recalling that Remdesivir is a delayed chain terminator (**Figure 2**). This implies that the Remdesivir-MP initially incorporated into the newly synthesized coronaviral RNA is the closest to the initial binding site of Remdesivir-TP in the coronaviral´s RdRp-RNA polymerase catalytic site. Finding at least one cluster of Remdesivir-TP overlapping with this Remdesivir-MP initially incorporated, confirmed that the docking procedure was optimal.

**Figure 3:**
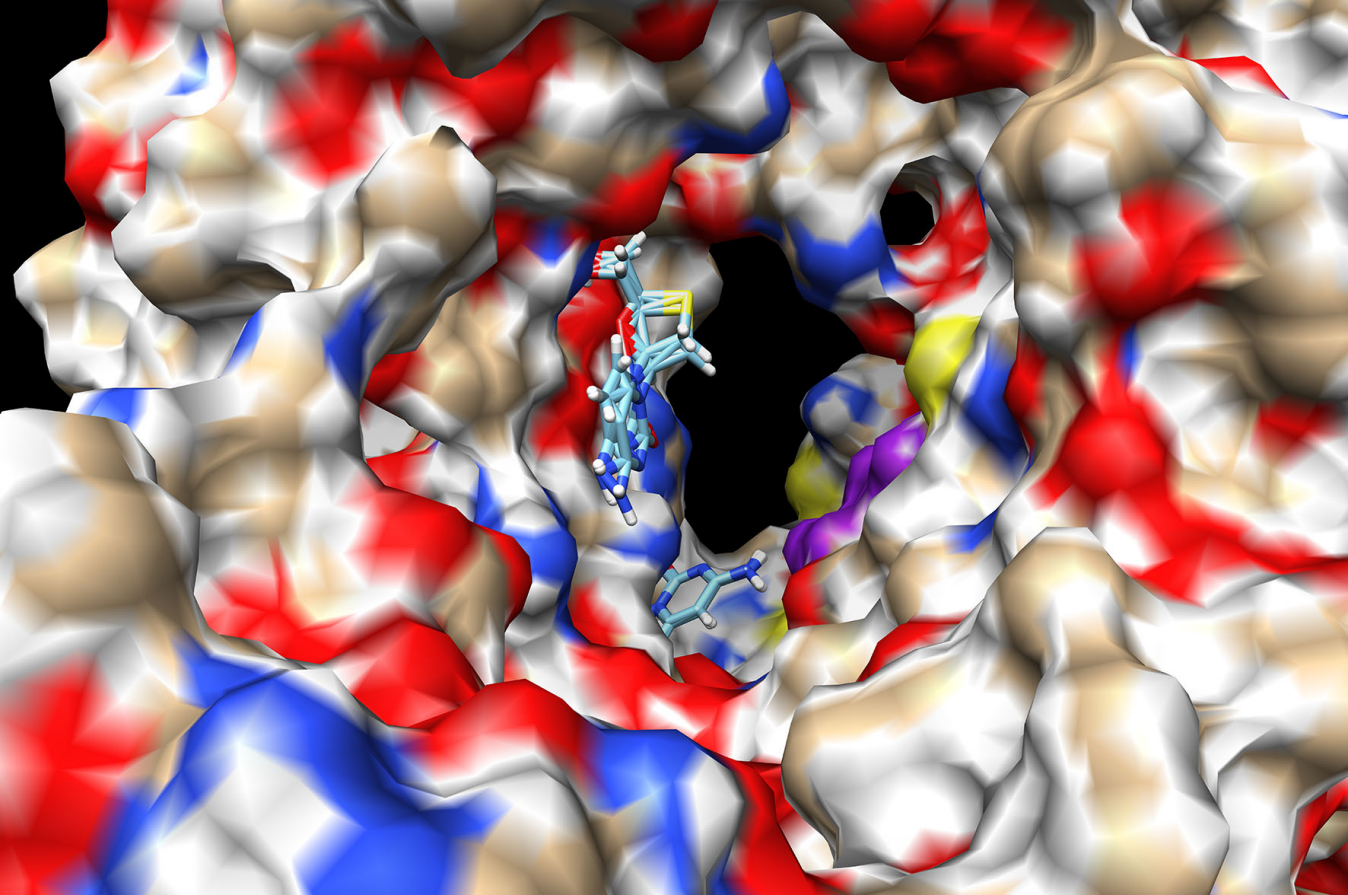
Docking of Lamivudine into the catalytic domain of the first structural model of SARS-CoV-RdRp. Two Lamivudine clusters (sticks) are bound to the catalytic site. The surface of the Chain A of the SARS-CoV-RdRp (PDB_id IO5S.pdb) is shown, with oxygen in red, nitrogen in blue, and aliphatic atoms in beige. Some conserved catalytic residues are depicted in purple. Both Lamivudine clusters are on close proximity to the catalytic residues in a position suitable for productive inhibition of RNA polymerization. The binding energy (ΔG°) is −9.05 kcal/mol for the more enriched 3-TC cluster (see **Table 1**). A more recent structure was used to improve the quality of this first docking analysis. Docking was carried out as described in the text, see anti-COVID-19 repurposing for details.

All dockings were carried out with the same procedure and conditions for all protein/nucleoside analogues or protein/nucleotide complexes. The results showed that Lamivudine docked at the catalytic site of a previous model (PDB_id IO5S.pdb) that contained a fragment of the chain A of the SARS-CoV (67) with a binding energy of −9.06 kcal/mol (**Figure 3** and **Table 2**), and an estimated *K*_d_ of 0.4 μM at the physiological temperature of 37°C, according to the equilibrium constant definition (ΔG°= −2.3RTlog(K)). To improve the docking analyses, in the course of the construction of a SARS-CoV-2-RdRp model, one cryo-EM structure of the SARS-CoV-2-RdRp was deposited in PDB with the PDB_id 6M71.pdb. This structure corresponds to the RNA polymerase of the actual COVID-19 pandemic and contained the main chain A or Nsp-12, with the catalytic and RNA polymerizing core and a couple of cofactors. Using this structure, a new successful Lamivudine docking analysis was carried out with chain A of the 6M71.pdb structure, since this one has the catalytic domain. This docking resulted in a binding energy of −6.89 kcal/mol, and an affinity estimated at 37°C of 1.4 x 10^−5^ M (see **Table 2**). Similar docking studies have shown that Lamivudine may also bind to the Receptor Binding Domain (RBD) of the Spike protein of SARS-CoV-2 (34), suggesting that in addition to binding to SARS-CoV-2-RdRp, the Lamivudine pro-drug could also inhibit the RBD–Spike-ACE2 interaction and therefore the binding and entrance of this coronavirus to the human cells.

**Table 2:** Docking of nucleoside/nucleotide analogs and nucleotides to the chain A of SARS-CoV-2 RdRp.

Docked molecule	ΔG°(kcal/mol)	Target (PDB_id)	${\text{K}}_{\text{a}}^{1}$ (M^−1^) at 37°C	${\text{K}}_{\text{d}}^{2}$ (M) at 37°C
Lamivudine	−9.06	IO5S.pdb^3^	2.40 × 10^+7^	4.17 × 10^−7^
Lamivudine	−6.89	6M71.pdb	7.11 × 10^+5^	1.40 × 10^−5^
Lamivudine-TP	−17.18	6M71.pdb	1.25 × 10^+12^	7.96 × 10^−13^
CTP	−13.58	6M71.pdb	3.65 × 10^+10^	2.73 × 10^−10^
Remdesivir	−6.86	6M71.pdb	6.77 × 10^+5^	1.47 × 10^−5^
Remdesivir-TP	−16.88	6M71.pdb	7.71 × 10^+11^	1.29 × 10^−12^
ATP	−13.10	6M71.pdb	1.68 × 10^+10^	5.95 × 10^−10^

1. Theoretical association constant (K_a_) calculated with the equation

ΔG° = −2.3RTlog(K) at 37°C.

2. Theoretical dissociation constant calculated as the inverse (1/x) of K_a_.

3. This structure corresponds to the first RdRp homology model of SARS-CoV.

**Figure 4:**
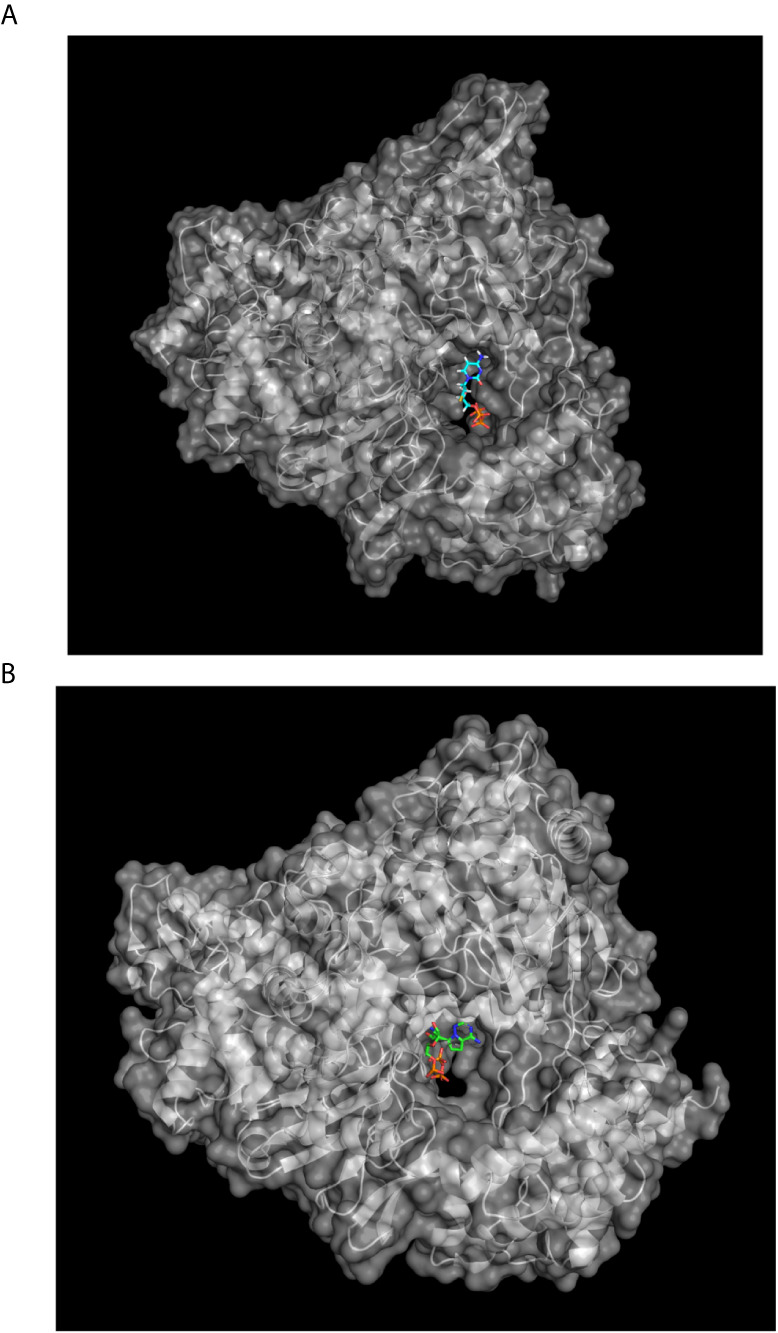
Lamivudine-TP docked to SARS-CoV-RdRp catalytic site and compared with the binding site of Remdesivir-MP in its cryo-EM structure. **(A)** The SARS-CoV-2-RdRp chain A (PDB_id 6M71.pdb) was prepared and used for docking of 3TC-TP as described in methods, the Lamivudine-TP molecule shown was that with the highest binding energy found in the catalytic site of the enzyme (see **Table 1**). The view corresponds to the catalytic site in the front right. **(B)** Remdesivir-MP (and PPi) bound to the SARS-CoV-2-RdRp-RNA complex, RNA is not shown for clarity but is bound to the catalytic site, Remdesivir is covalently linked to RNA.

**Figure 5:**
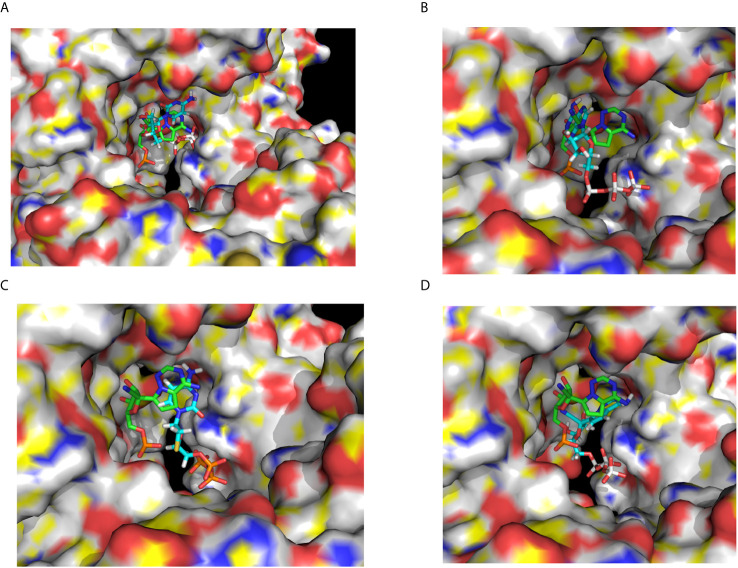
Docking of CTP, ATP, Lamivudine-TP and Remdesivir-TP to the SARS-CoV-2 RdRp. **(A)** Docking of CTP (blue) to the SARS-CoV-2 RdRp catalytic chain A superimposed to the remdesivir-MP observed by cryo-EM (green) **(B)** Docking of ATP (blue) to the SARS-CoV-2 RdRp chain A superimposed to the Remdesivir-MP observed by the cryo-EM (green) **(C)** Docking of Lamivudine-TP (blue) to the SARS-CoV-2 RdRp chain A superimposed to the remdesivir-MP observed by the cryo-EM (green) **(D)**. Docking of remdesivir-TP (blue) to the SARS-CoV-2 RdRp chain A superimposed to the Remdesivir-MP observed by cryo-EM (green). The images show a zoom-in from the same view shown in **Figure 4**, with the catalytic site in the front. The images correspond to some of the ligands enlisted in **Table 1**. In all cases the RdRp structure used for docking and shown here is the chain A of the SARS-CoV-2 RNA polymerase (PDB_Id 6M71.pdb).

To obtain a better docking analysis, the functional Lamivudine triphosphate (3TC-TP) molecule was docked to the SARS-CoV-2-RdRp chain A. For this purpose, the 3TC-TP molecule was extracted from the uploaded cryo-EM structure of the DNA polymerase complexed with DNA and 3TC-TP (PDB_id 6OUM.pdb), and prepared for docking as described above. Once more, at least one Lamivudine-TP overlapped with the binding site of the inhibitor Remdesivir-MP observed by cryo-EM (PDB_id 7BV2.pdb) and was bound with the highest binding energy (ΔG° = −17.18 kcal/mol) found for all ligands docked in this study (see **Figures 4**, **5**, and **Table 2**). At this site, Lamivudine-TP is positioned in productive binding to exert chain termination inhibition of the SARS-CoV-2-RdRp (see **Figure 5C**).

As a control for docking, CTP was also docked in the same catalytic site of the Chain A of the 6M71.pdb structure, overlapping with the bound Remdesivir-MP observed by cryo-EM (7BV2.pdb) (**Figure 5A**). The CTP cluster overlaps with the Remdesivir-MP, in the catalytic site docked with binding energies and affinities higher than those of 3TC. However, the theoretical *K*_d_ of binding of 3TC-TP at the catalytic site was about three orders of magnitude higher (7.96 x 10^−13^ M) than that of CTP (2.73 x 10^−10^ M), (see **Table 2**). This implies that Lamivudine-TP could be a very effective competitor with CTP for the catalytic site of SARS-CoV-2-RdRp, leading putatively to chain termination inhibition of the processive coronaviral RNA polymerase of the SARS-CoV-2-RdRp.

Finally, an important control was to carry out docking analysis in the same conditions with Remdesivir, but in the apo-RdRp chain A the 6M71.pdb structure as carried out here with Lamivudine, Lamivudine-TP, and CTP. For this purpose, the Remdesivir and Remdesivir-TP molecules were prepared for docking as described above. The docking of Remdesivir yielded one cluster at the catalytic site of the SARS-CoV-2-RdRp, overlapping with the cryo-EM-resolved Remdesivir-MP of the 7BV2.pdb structure. As expected, the pro-drug Remdesivir binds with relatively low affinity (ΔG° = −6.86 kcal/mol; *K*_d_ = 1.47 x 10^−5^ M), similar to that of the pro-drug Lamivudine (ΔG° = −6.89 kcal/mol; *K*_d_ = 1.40 x 10^−5^ M). If this trend is followed *in vivo*, Remdesivir may work as a weak ATP competitive inhibitor of the SARS-CoV-2-RdRp. However, as described with Lamivudine, Remdesivir will not exert chain termination inhibition, but only the Remdesivir-TP will be able to stall the SARS-CoV-2-RdRp (**Figure 2**). Accordingly, the docking of Remdesivir-TP was carried out and, as expected, the Remdesivir-TP docked at the catalytic site of the enzyme in the same position of the Cryo-EM–resolved Remdesivir-MP (see **Figure 5D**); this Remdesivir-TP binding took place with ΔG° = −16.88 kcal/mol and estimated *K*_d_ = 1.29 × 10^−12^ M, i.e. with about one order of magnitude lower affinity than that of Lamivudine-TP (ΔG° = -17.18 kcal/mol and *K*_d_ = 7.96 × 10^−13^ M). If these binding energies reflect the actual bindings *in vivo*, our proposed Lamivudine-TP could bind and inhibit the SARS-CoV-2-RdRp with similar or higher affinity than Remdesivir-TP, thus rendering Lamivudine-TP as a putatively potent inhibitor of the coronaviral RNA polymerase. One last control was also to carry out a docking of ATP on the NSP12, as this was necessary to find the relative affinity with which Remdesivir-TP will compete with ATP to inhibit the SARS-CoV-2-RdRp. In agreement with the previous dockings, the ATP docked at the catalytic site of the enzyme overlapping with the Remdesivir-MP observed by cryo-EM (see **Figure 5B**), with an estimated *K*_d_ of 5.95 × 10^−10^ and a ΔG° = −13.10 kcal/mol. This affinity was two orders of magnitude lower than that of Remdesivir-TP (*K*_d_ = 1.29 × 10^−12^), but similar to that of CTP (2.73 × 10^−10^) (see **Table 2**). Thus, as expected, the natural nucleoside triphosphate substrates CTP and ATP bind with similar affinities to the catalytic site of the SARS-CoV-2-RdRp, but Remdesivir-TP and Lamivudine-TP bind with higher affinities. It is worth noting that all dockings were carried out in the same conditions, i.e. we assume that their actual *K*_d_ values may be different *in vivo*, but the same ratios of *K*_d_´s may be kept *in vivo* as reported in **Table 2**.

It has been recently reported that Remdesivir does not seem to exert a clear therapeutic benefit for the recovery of COVID-19 patients overall in clinical trials [WHO (58)]. This may be because Remdesivir is a competitive inhibitor with ATP, which is the primary energy currency in all cells and living beings. This implies that Remdesivir competes with very high mM concentrations of ATP, thus may be unable to compete well with ATP because of the extremely high [ATP] in the cytosol. Therefore, other nucleoside/nucleotide analogues that compete with other nucleoside-triphosphates that are not the primary cellular energy currency such as CTP, may have better chances to successfully compete against the lower cytoplasmic [CTP] concentrations, as in the case of Lamivudine-TP. Taking into account these considerations, we suggest that it may be worth to assay further the effect of Lamivudine not only *in vitro*, with recombinant SARS-CoV-2 RdRp NSP-12 RNA polymerase activity as has been recently assayed (38), but also *in vivo*. The recent finding that Lamivudine can be incorporated and halt the SARS-CoV-2 RdRp or that it can induce mis-incorporations, i.e. mutations in the SARS-CoV-2 RdRp (38) supports the putative inhibitory action of Lamivudine on the SARS-CoV-2 RdRp. Although Lamivudine did not seem to affect SARS-CoV-2 replication in monkey and human cancerous cells *in vitro* (63), it was not analyzed whether Lamivudine is internalized and efficiently transformed into the active Lamivudine-TP in these cells as shown in **Figure 1B**. This is necessary because the efficiency of transformation of Lamivudine to Lamivudine-TP (**Figure 1**) may vary in different cell types (28). Therefore, we suggest that further analyses on the effect of Lamivudine and Lamivudine-TP should be carried out on the SARS-CoV-2 infection of non-cancerous human cells, as shown before (64) we could suggest the use of human MRC5 lung epithelial cells, a model the human lung epithelium *in vitro*. In the case that Lamivudine shows a clear inhibition of SARS-CoV-2 replication in MRC5 or other cultured human cells, the studies could proceed to SARS-CoV-2 infection in laboratory animals treated comparatively with Remdesivir and Lamivudine if needed, before considering Lamivudine for clinical trials in COVID-19 patients.

Regarding the specificity and side effects, Lamivudine seems to inhibit the mitochondrial DNA polymerase, or γ-DNA polymerase (36) putatively causing mitochondrial alterations in chronic hepatitis B patients (68). However, these side effects were observed with low statistical significance since the standard deviations of the mitochondrial parameters of Lamivudine *vs.* placebo-treated patients overlap statistically (68). Besides, these apparent side effects emerge during long-term prescription of Lamivudine. Again, some dietary supplements may overcome these side effects (69), and Lamivudine has been well tolerated during shorter treatments on hepatitis B patients (70, 71). Lamivudine has been prescribed for more than 25 years to HIV or HBV patients with very few cases of severe side effects, making it a relatively safe medication either prescribed as a single treatment or combined with other antivirals (29, 72). Thus it seems to be a well-tolerated drug in most cases, because it is a negative (−) enantiomer that inhibits specifically the viral but, in theory, not the human DNA or RNA polymerases (29). Furthermore, if Lamivudine-TP is able to inhibit the proof-reading activity of the human γ-mtDNApol as reported (73), thus it must also block the proof-reading activity of the coronaviral SARS-CoV-2-RdRp. This is supported by the report showing experimentally that Lamivudine-TP inhibits the RdRp of HCV virus (59), indicating that Lamivudine-TP must inhibit this 3´-5´exonuclease activity of coronaviral NSP-14, and also halt the SARS-CoV-2 replication. However, there are recent contradictory reports on the effect of Lamivudine on the SARS-CoV-2 RdRp, with inhibitory (38) and non-inhibitory (63) effects on SARS-CoV-2 replication. Our observed overlapping of the highest affinity docked 3TC-TP with the Remdesivir-MP resolved cryo-EM shown here (**Figure 5C**), suggests that Lamivudine-TP may inhibit the SARS-CoV-2-RdRp as a chain terminator as well as its 3’-5’ exonuclease activity, since the latter requires the ribose 2’-OH group (65) which is missing in Lamivudine (**Figures 1**, **2** and **Table 2**).

In theory, if lamivudine is an effective anti-COVID-19 agent, it should improve the patients’ outcomes when co-infected with HIV or HBV and SARS-CoV-2, and treated with 3TC. However, Lamivudine was withdrawn from treatments of HIV/COVID-19 patients upon hospitalization (74), even though most if not all of these patients had a positive outcome. For this reason, it is not possible to derive any conclusions on the putative effectiveness of Lamivudine against COVID-19 from that clinical study. However, a more recent survey study carried out with ≈ 500 HBV patients treated with or without Lamivudine, showed that the non-Lamivudine group had 48% of positive SARS-CoV-2 infected patients, and in contrast only 2% of the +Lamivudine group was positively infected with SARS-CoV-2 (75). Although this is a preliminary survey, it shows a clear and statistically different propensity to be SARS-CoV-2 positive in the absence of Lamivudine and to be SARS-CoV-2 negative in the +Lamivudine group (75). Another cohort study in Spain showed a lower incidence and severity of COVID-19 in HIV patients receiving Lamivudine and/or other ANNAs´ therapy vs. other HIV patients (76, 77). Of course these studies do not establish a direct cause-effect relationship between Lamivudine treatment and anti-SARS-CoV-2 inhibition in these HBV patients, but suggest that it may be worth assessing the putative inhibitory effect of Lamivudine by further *in silico*, *in vitro*, *in vivo*, and finally in clinical studies to try the prevention or treatment of COVID-19 with Lamivudine.

Lamivudine could also be combined with adjuvants of the immune response or other antivirals such as Remdesivir in case to be assayed for clinical COVID-19 studies (78). Furthermore, if as suggested from *in silico* dockings, Lamivudine also interferes with the RBD-Spike/ACE2 interaction *in vivo*, besides inhibiting the SARS-CoV-2 RdRp RNA polymerase, 3TC could have a good effectiveness against COVID-19. Another advantage of Lamivudine is that its patent was released several years ago, making it less expensive and thus more accessible than Remdesivir or Sofosbuvir (25, 26, 36, 37, 79, 80). Also, it is a controlled drug so it will not led to its self-medication by the COVID-19 patients.

In summary, we report here *in silico* data suggesting Lamivudine as a putative anti-COVID-19 medication. Similar docking studies led to useful proposals as in the case of Remdesivir-TP (25, 26). Comparing the binding energy of Sofosbuvir triphosphate (Sofosbuvir-TP) obtained by others (25, 26), it is much lower, i.e. −7.5 kcal/mol, than the one we obtained for Lamivudine-TP or 3TC-TP (−17.18 kcal/mol). These results suggest that Lamivudine or 3TC could be more effective as inhibitor of the SARS-CoV-2 RdRp RNA polymerase than Sofosbuvir, and other antivirals. In the course of preparing this review, some recent studies emerged examining the docking of the putative inhibitory binding of Lamivudine and other antivirals to the RdRp RNA Polymerase of SARS-CoV-2. These studies arrived at the same conclusion, i.e. that Lamivudine may bind with enough affinity to compete with CTP for the catalytic site of the SARS-CoV-2 RdRp, and putatively exert chain termination inhibition (81, 82). In one of these studies, Koulgi et al. (82) carried out docking experiments similar to ours; however, they found higher binding energies for ATP (−45.67 kcal/mol), CTP (−43.97 kcal/mol), Remdesivir-TP (−48.22 kcal/mol), and Lamivudine-TP (−23.7 kcal/mol) than those we found for ATP (−13.10 kcal/mol), CTP (−13.58 kcal/mol), Remdesivir-TP (−16.88 kcal/mol) and Lamivudine-TP (−17.18 kcal/mol) (**Table 2**). It may be difficult to explain the different binding energies observed between the study of Koulgi et al. (82), and our present results. However, there are three main differences between theirs and our docking studies that may account for the observed differences, namely, 1) the docking procedure since they allowed flexibility to the protein and ligands, whereas we only allowed flexibility for the ligand; 2) they used a different docking software, and 3) they used for docking the reduced structure of the RdRp SARS-CoV-2 RNA polymerase (PDB_id 7BTF.pdb), whereas we used the oxidized form of the same structure (PDB_id 6M71.pdb), both structures resolved in the same Cryo-EM study of Gao et al. (83). In summary, although both studies show different binding energy values, they converge in that Lamivudine may bind to the SARS-CoV-2 RdRp and putatively work as inhibitor this RNA polymerase. Furthermore, regardless of the differences between their binding energies and ours, the fact that the cells have higher mM [ATP] concentrations than [CTP] *in vivo*, because the former is the major energy currency of the cells, suggests that Lamivudine-TP must be able to compete with CTP for binding to the SARS-CoV-2 RdRp similarly, or possibly better than Remdesivir-TP competing *vs.* ATP for the same enzyme. In concordance with these conclusions, a recent review on the effect of ANNAs on the SARS-CoV-2 RdRp arrived at the same prediction for Lamivudine (84). Additionally, a recent docking report indicates the putative binding of Lamivudine to the main protease (MPro) of the SARS-CoV-2 (35), therefore it seems possible that, as reviewed here, Lamivudine could inhibit the three key SARS-CoV-2 processes: Spike/ACE2 interaction, processing by MPro and, as found here, RNA replication by the SARS-CoV-2 RdRp polymerase.

An important consideration is the emergence of resistant mutations of the SARS-CoV-2 against Lamivudine. In general, resistance to nucleoside analogues in retroviruses emerges after several months or years of treatment. However the relatively slow mutational rate of coronaviral RNA thanks to its proof-reading activity, makes very unlikely that coronaviral resistance to 3TC-TP might appear during a short treatment (weeks or a few months) with Lamivudine. It is worth recalling that this work does not discard the fact that other ANNAs may be also more or less effective against SARS-CoV-2. Further basic and clinical research is needed to confirm if Lamivudine and other antivirals are effective as inhibitors of SARS-CoV-2-RdRp activity *in vitro*, and as novel efficient therapies for COVID-19 patients.

## Concluding Remarks

In summary, we found that Lamivudine might be repurposed as both an anti-cancer drug, since it improves the outcome of radiotherapy and chemotherapy treated cancer patients. It also selectively diminishes the growth of cancerous cells *in vitro* compared with its lack of effect in the growth of non-cancerous control cells’ growth. Lamivudine (3TC) can also prevent the reactivation of Hepatitis Virus in cancer patients receiving radiotherapy and chemotherapy, thus providing a further protection to those patients with HBV and cancer. Furthermore, new clinical studies with Lamivudine, either combined or not with other ANNAs, show a significant improvement in cancer patients’ outcomes regardless of the primary anti-cancer treatment and the timing of Lamivudine-treatment onset. Another advantage of Lamivudine and related ANNAs is that 3TC may prevent the development of Hepato Cellular Carcinoma (HCC) by preventing the chronic HBV and cirrhosis that precedes the development of HCC. This prophylactic effect of Lamivudine and related ANNAs is progressively more accepted and novel delivery technologies are being applied to enhance the anti-viral and anti-cancer effectiveness of these ANNAs.

On the other hand, Lamivudine or 3TC may also be assessed as a putative anti-SARS-CoV-2 and anti-COVID-19 therapeutic drug. It may be docked in the same position where Remdesivir-TP binds at the catalytic SARS-CoV-2-RdRp site to putatively exert chain termination to inhibit SARS-CoV-2 replication. Together with other docking and emerging experimental studies, the overall results suggest that Lamivudine should be assessed along with ANNAs as an anti-SARS-CoV-2 agent. In summary further *in vitro* and *in vivo* assays are now needed to confirm whether this compound may or may not be useful in isolated or combined forms against the SARS-CoV-2 RdRp.

In sum, taken together and as reviewed here, all these studies confirm that it is worth including Lamivudine in the list of repurposed anti-cancer drugs because this repurposing against cancer is well established. On the other hand, although the anti-SARS-CoV-2 putative properties of Lamivudine still await for further experimental confirmation, the data available until now allow us to suggest repurposing Lamivudine as a putative treatment to assay *in vitro*, *in vivo*, and possibly in clinical trials against the SARS-CoV-2 RdRp and the COVID-19 pandemic. The possibility that other ANNAs different to Lamivudine might work against cancer and/or COVID-19 is not discarded here, instead this review open new windows for further theoretical and/or experimental studies to come. Hopefully this and the oncoming studies in these fields will call the attention of biomedical researchers and clinical authorities to assess if Lamivudine or related ANNAs may contribute to provide a better life quality and outcomes to cancer and/or COVID-19 patients.

## Author Contributions

JJG-T, RO, and MZ-Z wrote the manuscript and prepared figures and tables. All authors contributed to the article and approved the submitted version.

## Funding

This work was supported by the UNAM-DGAPA-PAPIIT grant project IN-217520.

## Conflict of Interest

The authors declare that the research was conducted in the absence of any commercial or financial relationships that could be construed as a potential conflict of interest.
